# Efficacy and safety of radiotherapy/chemoradiotherapy combined with immune checkpoint inhibitors for locally advanced stages of esophageal cancer: A systematic review and meta-analysis

**DOI:** 10.3389/fonc.2022.887525

**Published:** 2022-08-03

**Authors:** Jing Wu, Rong Deng, Tingting Ni, Qin Zhong, Fei Tang, Yan Li, Yu Zhang

**Affiliations:** ^1^ Department of Oncology, Guizhou Provincial People’s Hospital, Guizhou Cancer Center, Guiyang, China; ^2^ NHC Key Laboratory of Pulmonary Immune-related Diseases, Guizhou Provincial People’s Hospital, Guiyang, China

**Keywords:** immune checkpoint inhibitors, radiation therapy, esophageal cancer, efficacy, safety, meta-analysis

## Abstract

**Background:**

Radiotherapy (RT)/Chemoradiotherapy (CRT) are important treatments for all stages of esophageal cancer (EC). The combination of immune checkpoint inhibitors (ICIs) with RT/CRT seems to be promising avenue for the treatment of EC. Therefore, a systematic review and meta-analysis was performed in order to assess the safety and efficacy of RT/CRT and ICI combination therapy for EC patients.

**Methods:**

PubMed and several other databases were searched (according to specific criteria) to find relevant studies published prior to the 31^st^ of December 2021.

**Results:**

1962 articles were identified for screening, and six trials containing 668 patients were identified and pooled to determine the one- and two-year overall survival (OS), which were 84.5% (95% confidence interval (CI): 69.9%-100%) and 68.3% (95% CI: 49.0%-95.1%), respectively. Additionally, the rate of pooled grade 3-5 adverse reactions was 41.0% (95% CI: 31.2%-51.2%). The rate of specific grade 3-5 adverse reactions are as follows: lymphopenia (36.8%-60%), esophagitis (20%), anastomotic leakage (18%), esophageal fistula (10%), pain (10%), leukopenia (5.3%-10%), esophageal hemorrhage (2.5%-5%), chyle leakage (3%), fatigue (5%), cough (2.7%-5%), diarrhea (2.7%), pulmonary embolism (2.5%) and allergic reaction (2.5%). The pooled rate of pneumonitis of grade 3-5 and grade 1-5 was 0.8% (95% CI: 0.1%-0.16%, I^2^: 0%) and 5.4% (95% CI: 2.0%-14.2%, I^2^: 82%). For thoracic complication, esophagitis was 63.6% (95% CI: 42.4%-80.6%), which appeared to be more frequent with the combination of ICIs to RT/CRT (12%-37.7%). Other thoracic complications include esophageal hemorrhage (2.5%-10%), esophageal fistula (6%-10%) and anastomotic leakage (6%-21%). Additionally, some of the trials did not report cardiac related adverse reactions. The subgroup analyses also revealed that the pooled rate patients with grade 3-5 pneumonitis was higher for CRT/RT with concurrent and sequential ICI treatment (1.9%) than other groups (0.8%).

**Conclusion:**

This study suggests that the addition of ICIs to RT/CRT for EC patients may be both safe and feasible. However, larger randomized studies are needed to confirm these results.

## 1 Introduction

Esophageal cancer (EC) ranks seventh among all malignant tumors in terms of morbidity. It was also the sixth leading cause of cancer related death worldwide due to its aggressive nature (1). At present, the prognosis for EC is relatively poor and is predominantly treated with surgery, radiotherapy and chemotherapy (2). Moreover, limited progress has been made in the treatment of advanced esophageal cancer, for which the prognosis remains poor with a five-year survival rate of 5% for stage IV EC cases (3).

Radiation therapy (RT)/chemoradiotherapy (CRT) has been an important treatment in all stages of EC. Since the 1990s, the Radiation Therapy Oncology Group (RTOG) 85-01 trial indicated that chemoradiotherapy should be the standard care for unresectable, locally advanced EC (4). This notion was also supported by the Chemoradiotherapy for Oesophageal Cancer Followed by Surgery Study (CROSS), Shapiro et al. concluded that neoadjuvant CRT (compared with surgery alone) could improve survival for resectable EC (5).

Immunotherapy has rapidly become one of the most promising sources of novel anti-cancer drugs. It has considerably improved the prognosis for patients with various types of cancers. Immune checkpoint inhibitors (ICIs) enable the reversion of T cell suppression and enhance anti-tumor immune responses by blocking programmed cell death 1 (PD-1, PDCD1)/programmed death-ligand 1 (PD-L1, CD274) signaling (6). Numerous ICI clinical trials have reported promising anti-tumor activity of for the treatment of EC (7–9). Some attention has been given to the clinical efficacy and safety of combination RT/CRT and ICIs for EC, but further investigation is still needed (10–15).

This is particularly important given the lack of consensus on the utility of RT/CRT and ICIs combination therapy for other cancers. Some studies have demonstrated that RT/CRT plus ICIs enabled an increased anti-tumor efficacy for non-small cell lung cancer (NSCLC) (16, 17). However, Cho et al. reported that RT-induced lymphopenia reduced the efficacy of ICIs (18). Additionally, other studies have observed increased toxicity (especially pulmonary toxicity) when ICIs were used in combination with RT/CRT (19, 20). Pre-clinical data suggested that RT generated oxidative damage to DNA and proteins in lung tissue, causing pulmonary injuries. This contributes to the release of tumor antigens and inflammatory factors, which activate T cells. ICIs also activate T cells and promote inflammation, which may damage otherwise healthy tissues when used the combination with RT. Thereby exacerbating pulmonary toxicity in addition to the amplification of anti-tumor effects (21, 22). The *post hoc* analysis from the phase I KEYNOTE-001 trial noted a significant increase in combination treatment related pulmonary toxicities (13% vs. 1%, *P*=0.046), and a borderline increase in the incidence of all pulmonary toxicities (63% vs. 40%, *P*=0.052), including dyspnea, cough, pneumonitis, and respiratory failure (23). Botticella et al. also observed the occurrence of grade ≥ 3 pneumonitis in 16.7% of the patients receiving combination therapy compared to 2.4% of the patients receiving ICIs alone (*P*=<0.001) (24). Moreover, the use of this combination therapy in trials may not reflect the real-world data. For example, patients with a history of interstitial lung disease (ILD) were excluded from the aforementioned trials. Indeed, Suresh et al. found a higher incidence of immune-associated pneumonia in real-world setting (25). For these reasons, the efficacy and safety of RT/CRT and ICIs combination therapy remains controversial. Therefore, we performed a systematic review and meta-analysis herein to elucidate the safety and efficacy of RT/CRT and ICIs combination therapy for EC.

## 2 Materials and methods

### 2.1 Search Strategy and Selection Criteria

This systematic review and meta-analysis was conducted using a Preferred Reporting Items for Systematic Review (PRISMA) and Meta-analysis statement (26). Ethical approval was not required for this study because all the data is derived from previously published sources.

PubMed, ISI Web of Science and the Cochrane Library were searched to identify literature in English language journals. This study utilized articles published prior to the 31^st^ of December 2021, without a lower date boundary. The following search terms were used: 1) “o) esophageal neoplasm (s)/cancer (s)/carcinoma (s)/adenocarcinoma (s)/squamous cell carcinoma (s)” or “(o) esophagus neoplasm (s)/cancer (s)/carcinoma (s)/adenocarcinoma (s)/squamous cell carcinoma (s)” or “gastro esophageal neoplasm (s)/cancer (s)/carcinoma (s)” or “Barrett (s) (o) esophagus” or “(o) esophageal squamous dysplasia”. 2) “radiotherapy” or “radiation therapy” or “radiation treatment” or “radio-chemotherapy” or “chemoradiotherapy”. 3) “immunotherapy” or “immune checkpoint inhibitors” or “programmed cell death 1 receptor” or “programmed cell death 1 ligand 1” or “cytotoxic T lymphocyte-associated antigen-4 (CTLA-4) antigen” or “anti-CTLA-4” or “anti-PD-1” or “anti-PD-L1” or “Durvalumab” or “Atezolizumab” or “Pembrolizumab” or “Nivolumab” or “Toripalima” or “Tislelizumab” or “Camrelizumab” or “Sintilimab” or “Tremelimumab” or “Ipilimumab” or “PDCD1” or “CD274”.

Studies were included in the meta-analysis if they met the following criteria: 1) describe participants with histologically confirmed esophageal cancer; 2) immune checkpoint inhibitors were used in combination with radiotherapy or chemoradiotherapy, radiotherapy with sequential or concurrent ICIs therapy, or radiotherapy alone (including conventional radiotherapy and stereotactic radiotherapy); 3) utilized a prospective or retrospective study design; 4) outcomes included clinical efficacy and treatment safety; 5) published in English.

Conference abstracts, case reports, comments, reviews, studies in animals, and mechanistic studies were excluded. Studies without sufficient data (missing clinical outcomes data) or unclear descriptions (the description of the trial was not clear or have not precisely measured or described the outcomes of trial) were also excluded. When articles described the same study population, only the most recent or the most complete analysis was included. Disagreements related to article selection were resolved during group discussions with all the authors of this study.

### 2.2 Data extraction

The following data was extracted by two independent researchers: the first author’s name, time of publication, country, number of cases, position of the tumor, pathological subgroups, treatment regimens, radiotherapy type and dose, drugs used, anti-PD-1/PD-L1 therapy dosage, time of publication. Data related to the study outcomes was also extracted, such as the overall survival (OS), progression free survival (PFS) and the number of patients who experienced adverse reaction. Any discrepancies were resolved by group discussion until a consensus was reached. The revised Cochrane Risk of Bias tool for randomized trials (RoB2) (27) and Risk of Bias in Non-randomized Studies of Interventions (ROBINS-I) tool (28) were used for the quality assessment of randomized controlled trials (RCTs) and non-randomized trials respectively.

### 2.3 Statistical analysis

A meta-analysis was performed using the random-effects model by “meta” package implemented in R (version 4.1.2, R Foundation for Statistical Computing). A 95% confidence interval (CI) was adopted. Heterogeneity among studies was assessed using Cochran’s Q and I^2^ statistics. I^2^ values of 0%, 25%, 50% and 75% representing no, low, moderate and high heterogeneity, respectively. A meta-regression was considered to be inappropriate due to the insufficient study volume (<10). Subgroup and sensitivity analyses were conducted to examine sources of study heterogeneity and determine the influence of each individual study. The possibility of publication bias was estimated using the Begg’s and Egger’s test. A threshold of *P=*<0.05 was used when considering the statistical significance.

## 3 Results

**Figure 1:**
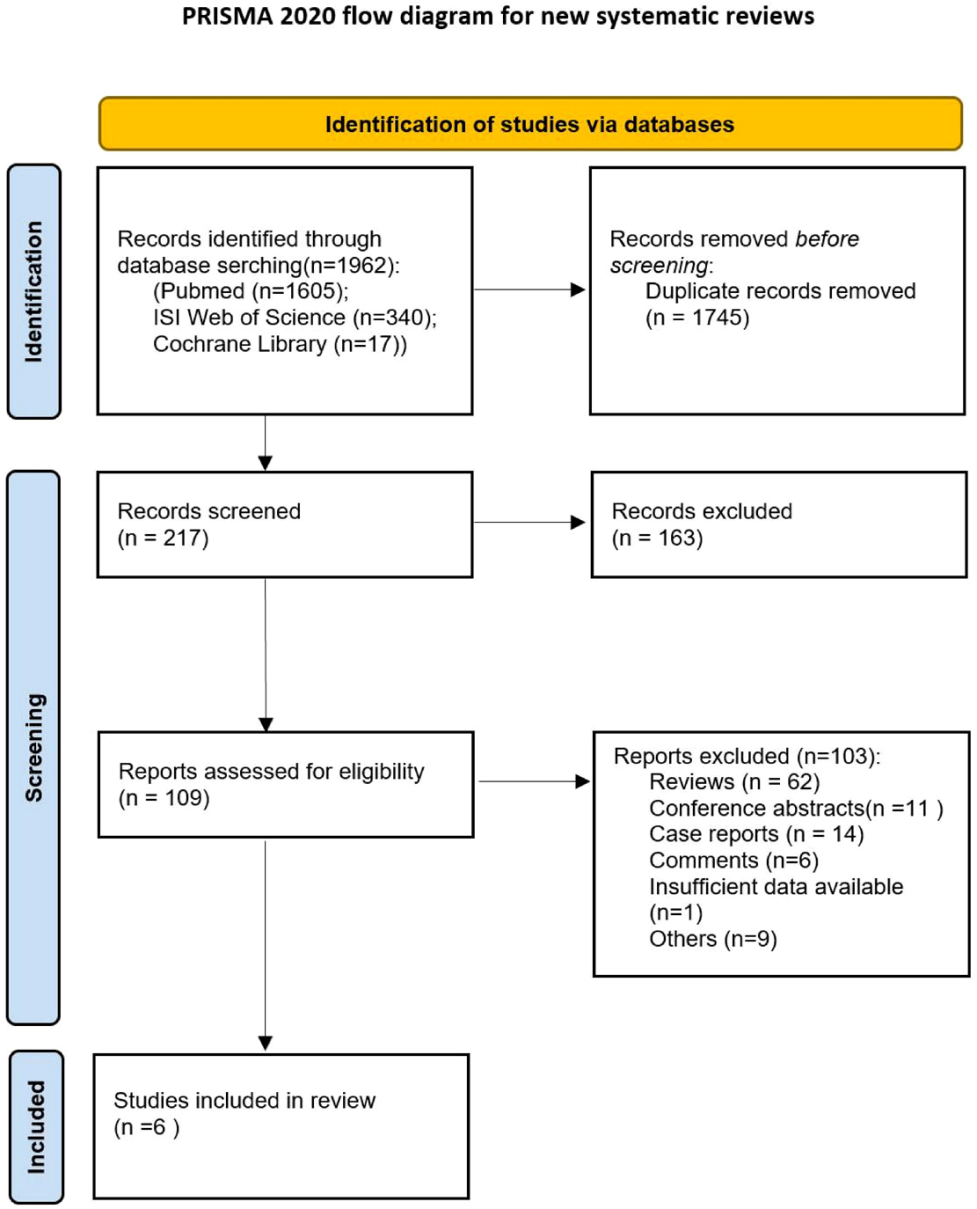
Flow diagram of included and excluded studies.

The systematic study search process (**Figure 1**) enabled the identification of six trials (**Table 1**) for this systematic review and meta-analysis (10–15). The six trials consisted of five non-randomized trials (10–13, 15) and one randomized controlled trial (14). Two trials were conducted in the United States of America (14, 15), three in China (10, 12, 13), and one in the Netherlands (11). Of the six studies included, two were phase I/Ib trials (12, 13), three were phase II trials (10, 11, 15), one was a phase III trial (14). The drug camrelizumab was used in two studies (12, 13), whereas pembrolizumab (10), durvalumab (15), atezolizumab (11) and nivolumab (14) were used in one study each. ICIs were administered after CRT in two studies (14, 15), concurrently with CRT in one study (10); whereas three studies examined both concurrent and sequential administration of CRT/RT with ICIs (11–13). ICIs were administered for less than six months in two trials (10, 11), up to 12 months in two trials (14, 15) and up to 32 weeks in two other trials (12, 13). The total radiation dose was 41.4 Gy in two studies (10, 11) and 60 Gy in two other studies (12, 13). The RCT (14) was deemed to be at low overall risk of bias. Three non-randomized trials (10, 12, 13) were judged to be at moderate risk of bias, and two (11, 15) had a low overall risk of bias (**Supplementary Materials Figures 1**–**4**). A total of 668 patients were included in the aforementioned trials.

**Table 1:** Main characteristics of the selected studies.

Study	Year	Trial Phase	Country	N	Cancer Type		Treatment	ICI Dose	Intervention Period	OS	PFS	Toxicity ≥Grade 3 (%)
Hirva Mamdani	2021	II	US	37		rE/rGEJ AC	Sequential. Neoadj CRT: - standard RT dose - FP or TC QW Surgery: -R0 resection (Pathological residual tissue)	Durvalumab 1500 mg IV Q4W	13 doses (12 months), or until unacceptable toxicities or disease recurrence.	NR	NR	10 (27%)
Kelly	2021	III	US	532		rE/rGEJ	Sequential. Neoadj CRT: <41.4Gy 12% <40Gy 1% 40-41.4Gy 11% 41.4-50.4Gy 64% >50.4 Gy 18% NA 6% TC 73% FP 14% Other 12% Surgery: -R0 resection (Pathological residual tissue)	Nivolumab 240 mg IV Q2W- 480mg IV Q4W	240 mg every 2 weeks for 16 weeks, followed by 480 mg every 4 weeks (12 months)	mOS 22.4 months	NR	183 (34%)
Zhang	2021.06	I/Ib	CN	19		Locally advanced ESCC	Concurrent and Sequential. RT: - 60 Gy (2.0 Gy/fraction)	Camrelizumab 200 mg IV Q2W	From radiotherapy onset for up to 32 weeks	mOS 16.7 months	mPFS 11.7 months	10 (53%)
Zhang	2021.09	1b	CN	20		Locally advanced ESCC	Concurrent and Sequential. CRT: - RT: 60 Gy (2.0 Gy/fraction) - DP QW DTX 25mg/m^2^ CDDP 25mg/m^2^	Camrelizumab 200 mg IV Q2W	From radiotherapy onset for up to 32 weeks	1-year OS: 85% 2-Year OS: 69.6%	1-year PFS: 80% 1- Year PFS: 69.6%	9 (45%)
Li	2020	II	CN	20		rESCC	Concurrent. Neoadj CRT: - RT: 41.4 Gy (1.8 Gy/fraction) -TC QW: CBDCA AUC=2 PTX 50mg/m^2^ Concurrent and Sequential. Surgery: -Within 4-6 weeks post-surgery	Pembrolizumab 2mg/kg IV Q3W	2 doses	NR	NR	13 (65%)
Ende	2021	II	NL	40		rEAC	Neoadj CRT: - RT: 41.4 Gy (1.8 Gy/fraction) -TC QW: CBDCA AUC=2 PTX 50mg/m^2^ Surgery: -Within 14-16 weeks post-surgery	Atezolizumab 1200 mg IV Q3W	5 doses	mOS 29.7 months	mPFS 19.4 months	16 (40%)

N, Number of patients; ICIs, Immune checkpoint inhibitors; OS, Overall survival; PFS, Progression free survival; rE, Resectable esophageal cancer; rGEJ, Resectable gastroesophageal junction cancer; AC, Adenocarcinoma; Neoadj, Neoadjuvant; CRT, Chemoradiotherapy; RT, Radiotherapy; FP, 5-Fluorouracil plus cisplatin; TC, Paclitaxel plus carboplatin; QW, Once a week; IV, Intravenous; Q4W, Every 4 weeks; NR, Not reported; ESCC, Esophageal squamous cell carcinoma; Q2W, Every 2 weeks; mOS, Median overall survival; mPFS, Median progression-free survival; rESCC, Resectable esophageal squamous cell carcinoma; CBDCA, Carboplatin; AUC, Area under the curve; PTX, Paclitaxel; Q3W, Ever 3 weeks; DP, Docetaxel plus cisplatin; DTX, Docetaxel; CDDP, Cisplatin; rEAC, Esophageal adenocarcinoma.

### 3.1 Efficacy

The trials included in this study had different primary efficacy variables, which prevented the performance of an efficacy based meta-analysis. The pathological complete response (pCR) rate was 30.3-55.6% for patients treated with neoadjuvant CRT and ICIs (10, 11), with a major pathological response (mPR) of 89% (10), and a 94%-100% R0 resection rate (10, 11). For locally advanced EC cases, the rate of complete remission (CR) was 10%-10.5% (12, 13), partial remission (PR) was 55%-63.2% (12, 13), with an objective response rate (ORR) of 65%-73.7% (12, 13).

### 3.2 Survival

**Figure 2:**
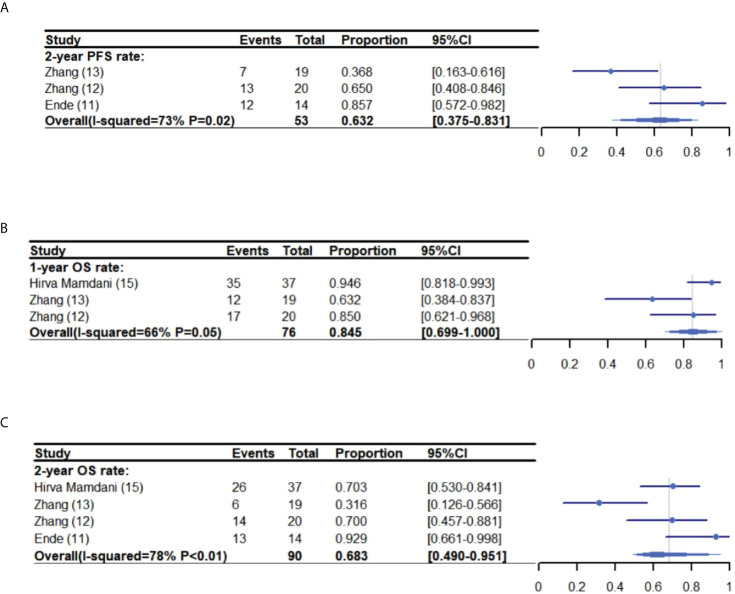
One-year PFS meta-analysis and forest plot **(A)**, one-year OS **(B)** and two-year OS **(C)** for esophageal cancer patients treated with ICIs and CRT/RT. PFS, Progression free survival; OS, Overall survival; ICIs, Immune checkpoint inhibitors; CRT, Chemoradiotherapy; RT, Radiotherapy.

The pooled two-year PFS was 63.2% (95% CI: 37.5%-83.1%, I^2^: 73%) (11–13), with a one-year and two-year OS of 84.5% (95% CI: 69.9%-100%, I^2^: 66%) (12, 13, 15) and 68.3% (95% CI: 49.0%-95.1%, I^2^: 78%), respectively (11–13, 15) (**Figure 2**).

Mamdani, et al. (15) reported that patients who received sequential CRT and ICIs treatment had a one-year recurrence free survival rate (RFS) of 73%, a two-year RFS of 51.4%. Whereas, Kelly, et al. (14) reported a one-year disease-free survival rate (DFS) of 62%, and a one-year distant metastasis free survival rate (DMFS) of 92.1%. For locally advanced EC, Zhang, et al. reported the rate of locoregional recurrence-free survival as 62.7% at 12 months and 48.8% at 24 months (13). Additionally, Zhang, et al. found that the one-year PFS was 47.4%-80% (12, 13).

### 3.3 Grade 3-5 adverse reactions

**Figure 3:**
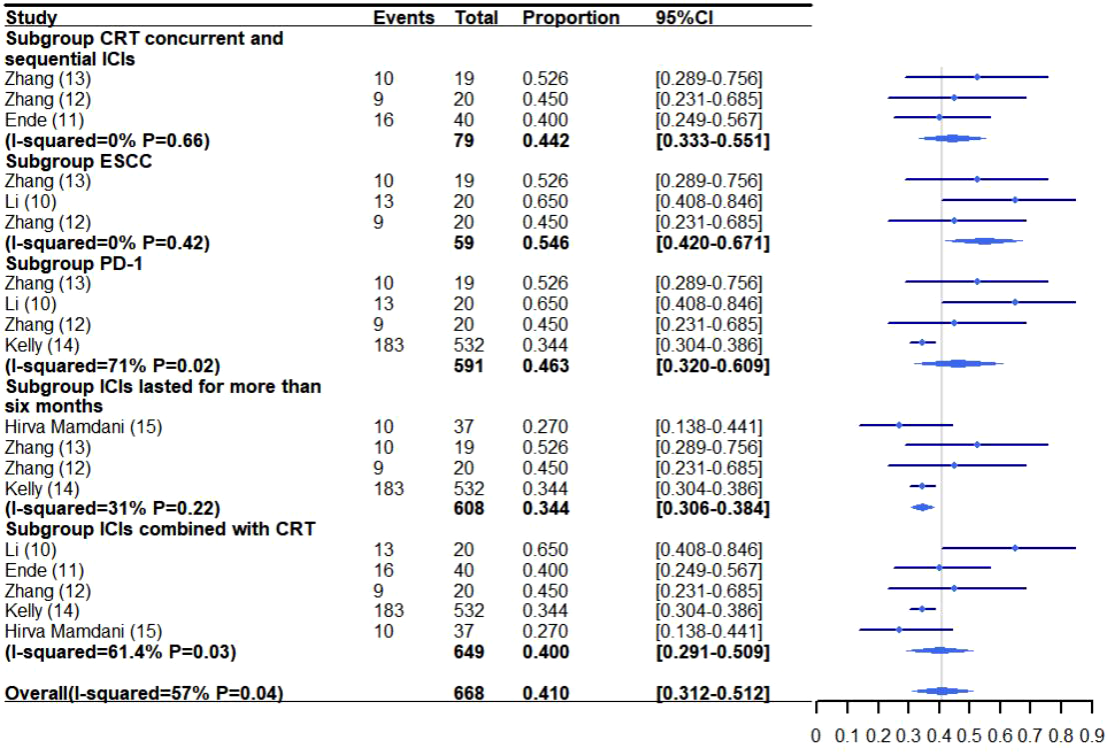
Grade 3-5 adverse reaction meta-analysis and forest plot for esophageal cancer patients treated with CRT/RT and ICIs. CRT, Chemoradiotherapy; RT, Radiotherapy; ICIs, Immune checkpoint inhibitors; ESCC, Esophageal squamous cell carcinoma; PD-1, Programmed death-1.

The pooled rate of grade 3-5 adverse reactions from all the studies was 41.0% (95% CI: 31.2%-51.2%, I^2^: 57%) (10–15) (**Figure 3**). The pooled rate of the other grades were not analyzed due to excessive heterogeneity, with few trials reporting related side effects. The rate of specific adverse reactions are as follows: 36.8%-60% of patients experienced Lymphopenia (10, 13), 20% radiation esophagitis (12), 18% anastomotic leakage (11), 10% esophageal fistula (12), 10% pain (12), 5.3%-10% leukopenia (10, 12, 13), 2.5%-5% esophageal hemorrhage (10, 11), 3% chyle leakage (11), 5% fatigue (12), 2.7%-5% cough (10, 12, 13, 15), 2.7% diarrhea (15), 2.5% pulmonary embolism (11) and 2.5% allergic reaction (11).

### 3.4 Pneumonitis and cough

**Figure 4:**
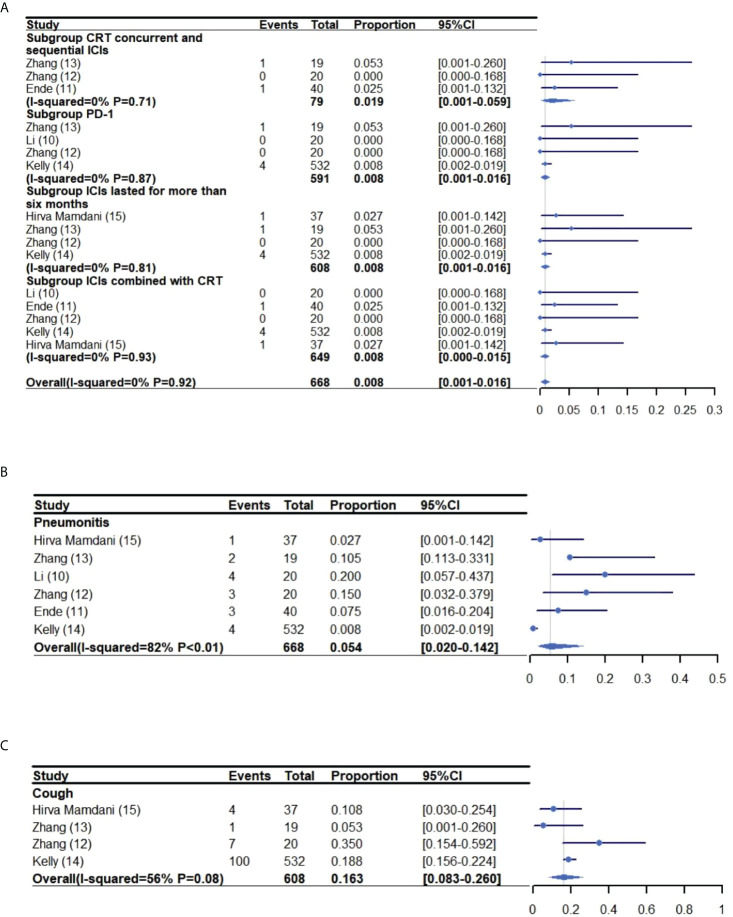
Meta-analysis and forest plot for esophageal cancer patients treated with CRT/RT and ICIs who experienced grade 3-5 Pneumonitis **(A)**, grade 1-2 pneumonitis **(B)** and cough **(C)**. CRT, Chemoradiotherapy; RT, Radiotherapy; ICIs, Immune checkpoint inhibitors; PD-1, Programmed death-1.

The pooled rate of grade 3-5 and grade 1-5 pneumonitis was 0.8% (95% CI: 0.1%-0.16%, I^2^: 0%) (10–15) and 5.4% (95% CI: 2.0%-14.2%, I^2^: 82%) (10–15), respectively. The incidence of grade 1-5 cough was 16.3% (95% CI: 8.3%-26.0%, I^2^: 56%) (12–15) (**Figure 4**).

### 3.5 Thoracic complication

**Figure 5:**
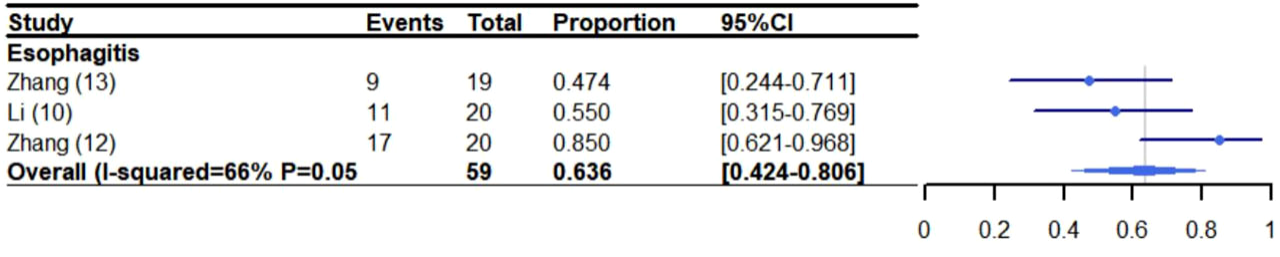
Meta-analysis and forest plot for patients who experienced esophagitis receiving CRT/RT and ICIs. CRT: Chemoradiotherapy; RT: Radiotherapy; ICIs: Immune checkpoint inhibitors.

The incidence of esophagitis was 63.6% (95% CI: 42.4%-80.6%, I^2^: 66%) (10, 12, 13) (**Figure 5**). Other thoracic effects were not assessed specifically because few trials reported these side effects. However, some thoracic effects were reported as follows: anastomotic leakage (6.0%-21.0%) (10, 11), chyle leakage (15.0%) (11), esophageal fistula (6%-10%) (10, 12), pulmonary embolism (8%) (12), esophageal stenosis (5%) (11, 12), esophageal hemorrhage (2.5%-10%) (10, 11) and chylothorax (2.5%) (11). Additionally, some of the trials did not report cardiac related adverse reactions.

### 3.6 Other adverse reactions

A pooled meta-analysis of other adverse reactions was presented in **Supplementary Materials Figure 5**. 16.1% (95% CI: 13.2%-16.1%, I^2^: 0%) of patients experienced vomiting (10, 11, 14), 22.6% (95% CI: 13.7%-32.9%, I^2^: 0%) constipation (11–13), 16.7% (95% CI: 13.8%-19.7%, I^2^: 0%) diarrhea (10, 11, 14, 15), 0.9% (95% CI: 0.0%-3.1%, I^2^: 33%) gastric bleeding (10, 11, 14), (95% CI: 7.6%-12.5%, I^2^: 8%) hypothyroidism (12–15) 9.7% and 9.0% (95% CI: 6.7%-11.5%, I^2^: 0%) skin rash (11, 14, 15).

### 3.7 Grade 3-5 adverse reaction subgroups

#### 3.7.1 Subgroup analysis of grade 3-5 adverse reactions following CRT/RT with concurrent and sequential ICIs treatment

A subgroup analysis was performed to investigate the incidence of grade 3-5 adverse reactions for patients receiving concurrent and sequential CRT/RT and ICI treatment (11–13) (**Figures 3**–**4A**). The rate of grade 3-5 adverse reactions was 44.2% (95% CI: 33.3%-55.1%, I^2^: 0%) and grade 3-5 pneumonitis was 1.9% (95% CI: 0.1%-5.9%, I^2^: 0%).

#### 3.7.2 Subgroup analysis of grade 3-5 adverse reactions

The incidence of grade 3-5 adverse reactions for ESCC patients treated with combination therapy was reported in three trials (10, 12, 13) (**Figures 3**–**4A**). The pooled rate of grade 3-5 adverse reactions was 54.6% (95% CI: 42.0%-67.1%, I^2^: 0%).

#### 3.7.3 Subgroup analysis of grade 3-5 adverse reactions of PD-1

The rate of grade 3-5 adverse reactions in patients receiving CRT/RT and PD-1 inhibitors was 46.3% (95% CI: 32.0%-60.9%, I^2^: 71%) and 0.8% (95% CI: 0.1%-1.5%, I^2^: 0%) for grade 3-5 pneumonitis (10, 12–14) (**Figures 3**–**4A**).

#### 3.7.4 Subgroup analysis of grade 3-5 adverse reactions to ICIs that lasted for more than six months

ICIs were administered for more than six months in four trials (12–15). The pooled incidence of grade 3-5 adverse reactions was 34.4% (95% CI: 30.6%-38.4%, I^2^: 31%) and grade 3-5 pneumonitis was 0.8% (95% CI: 0.1%-1.6%, I^2^: 0%) (**Figures 3**–**4A**).

#### 3.7.5 Subgroup analysis of grade 3-5 adverse reactions of CRT combined with ICIs

The rate of grade 3-5 adverse reactions in patients receiving CRT and ICIs was 40.0% (95% CI: 29.1%-50.9%, I^2^: 61.4%) and 0.8% (95% CI: 0.1%-1.5%, I^2^: 0%) for grade 3-5 pneumonitis (10–12, 14, 15) (**Figures 3**–**4A**).

### 3.8 Publication bias

There was some publication bias for the of grade 3-5 pneumonitis (ESCC) subgroup analysis. So, the grade 3-5 pneumonitis subgroup analysis was not conducted. The Begg’s and Egger’s tests found that there was no publication bias in the other analyses (**Supplementary Materials Table 1**).

## 4 Discussion

The study contained herein provides an overview of published trials that focus upon the use of RT/CRT with ICIs in EC patients. This review and meta-analysis, systematically, quantitatively and comprehensively analyzes the clinical efficacy and safety of RT/CRT when combined with ICIs for the treatment of EC. However, it does have some limitations that are mostly related to the availability of data/studies in this field. This prevented the exploration of some details surrounding the efficacy and safety of RT/CRT plus ICIs (such as the influence of different types of chemotherapeutics, radiotherapy doses, fractions, and target volumes. Additionally, most of the studies included in the meta-analysis were single arm clinical trials, which prevents a comparison between the advantages and disadvantages of CRT/RT with ICIs and CRT/RT based upon a balanced baseline.

The primary efficacy variable published by each study was also different. Therefore, the analysis could only cover the one-year OS (84.5%: 95% CI: 69.9%-100%, I^2^: 66%), two-year OS (60.0%: 95% CI: 41.2%-87.5%, I^2^: 62%) and two-year PFS (63.2%: 95% CI: 37.5%-83.1%, I^2^: 73%). Nevertheless, this enabled the estimation that the 2-year OS rate was 36.4%-61.5% for patients with locally advanced EC patients treated CRT (29–32). EC patients treated neoadjuvant CRT and ICIs had a pCR rate of 30.3% in the PERFECT trial (11) and 55.6% in the PALACE-1 trial (10). The pCR rate was higher in PALACE-1 trial than that reported by the other trials, which only administered neoadjuvant CRT (33–36). Except for the CheckMate-577 trial, which evaluated the adjuvant use of nivolumab for patients that were administered neoadjuvant CRT and patients post resection with residual pathologic tissue. In 2021, the US Food and Drug Administration (FDA) (37) and the European Medicines Agency (EMA) (38) approved the use of nivolumab for EC patients experiencing disease progression following CRT, which was associated with superior DFS when compared with a placebo (median DFS, 22.4 vs. 11 months, HR 0.69; 95% CI, 0.56–0.86, *P=*<0.001).

Additionally, this meta-analysis suggests that the rate of grade 3-5 adverse reactions was similar for patients receiving/RT and ICIs when compared to RT/CRT alone (27%-61.5%) (29, 33, 39, 40). The incidence of patients experiencing grade 3-5 adverse reactions ranged from 27% to 65%, with an overall rate of 41.0% (95% CI: 31.2%-51.2%) in the pooled analysis. The rate of grade 3-5 pneumonitis was 0.0%-5.3%, with a rate of 0.8% (95% CI: 0.1%-0.16%) in the pooled analysis. Cardiac related adverse reactions were not reported by the trials, with the exception of CheckMate 577 (14), which reported the death of one patient due to cardiac arrest. However, that event was not thought to be related to ICIs by the investigators. Therefore, it appears that the combination of RT/CRT with ICIs is a safe treatment option, although further study may be warranted.

It is notable that few trials reported esophageal related side effects, which prevented the performance of a pooled analysis. But, it appears that combined treatment did not increase the occurrence of esophageal hemorrhage (2.5%-10% vs 0.5-8%) (33, 34), esophageal fistula (6%-10% vs 1.1%–22%) (33, 41, 42) and anastomotic leakage (6%-21% vs 8.6-22%) (33, 34). Nevertheless, future trials should probably pay attention to the reporting of esophageal events, which as the site of this cancer could be at risk of perforation and bleeding. Indeed, one patient with grade III lymphopenia experienced significant esophageal hemorrhage after the second dose of chemotherapy and died while awaiting surgery (10). The NEOCRTEC5010 (33) and CROSS trials (43) also reported the death of patients due to esophageal hemorrhage. This notion is also supported by the pooled esophagitis analysis, since 63.6% (95% CI: 42.4%-80.6%, I^2^: 66%) (10, 12, 13) of patients experienced this event when administered an ICI and CRT combination, which was more frequent when compared to patients receiving CRT alone (12%-37.7%) (30, 33, 34, 44, 45). However, this finding should be interpreted with caution due to the relatively small number of cases reported.

The subgroup analyses revealed that the pooled rate of grade 3-5 pneumonitis was higher for patients treated with CRT/RT and concurrent/sequential ICIs (1.9%) when compared to patients receiving other regimens (0.8%). However, it remains unknown whether there are toxicity differences between concurrent and sequential administration of CRT/RT with ICIs for EC patients. This notion may require further investigation because Zhang, et al. (46) reported that patients treated with ICIs before or during thoracic radiotherapy developed radiation pneumonia at a higher rate than patients treated with ICIs after radiotherapy (27/45 vs 14/50, 60% vs 28%, *P*=0.01). Hence, EC patients may also experience an increased incidence of adverse reactions when treated with ICIs before or during. However, we also think the addition of ICIs to RT/CRT for EC patients may be both safe and feasible.

Esophageal adenocarcinomas (EAC) and esophageal squamous cell carcinomas (ESCC) have distinct histopathology, epidemiology, and molecular characteristics. A comprehensive molecular analysis showed that ESCC is more similar to squamous cell carcinoma located in other organs, whereas EAC is more similar to the chromosomal instability subtype of gastric cancer (47). Positive PD-L1 expression seems higher in ESCC than EAC (48–51). A meta-analysis evaluating the prognostic value of PD-L1 in ESCC showed a correlation of high PD-L1 expression with distant metastasis and poor OS (49). However, PD-L1 expression did not seem to affect survival in EAC (51). Interestingly, KEYNOTE-180 evaluated pembrolizumab in a third and further- setting for patients with advanced and metastatic esophageal cancer (52), the ORR in the whole population was 9.9% (95% CI, 5.2–16.7), 14.3% (95% CI, 6.7–25.4) among ESCC, and 5.2% (95% CI, 1.1–14.4) among EAC. Based on these positive results, the KEYNOTE-181 trial, investigating pembrolizumab versus chemotherapy in patients with advanced/metastatic SCC or AC of the esophagus, which progressed after one prior therapy session, was initiated. Although pembrolizumab showed promising results in the overall cohort, the most significant benefit was seen in ESCC (8.2 versus 7.1 months; HR 0.78; 95% CI 0.63–0.96; p = 0.0095) (53). Additionally, the combination of the CROSS regimen with adjuvant nivolumab in the aforementioned CheckMate 577 (14) trial showed a greater disease-free survival benefit in the ESCC subgroup (AC: placebo 11.1 months (95% CI 8.3–16.8) versus nivolumab after 19.4 months (95% CI 15.9–29.4); SCC: placebo 11.0 months (95% CI 7.6–17.8) and versus nivolumab after 29.7 months (95%CI 14.4–not estimated).

These findings indicate that the combination of systemic and radiotherapy acts by sensitizing SCC cells, and thereby leads to a major survival benefit compared to other strategies. Three of the studies included herein explored the efficacy and adverse events associated with RT/CRT and ICIs combination therapy for esophageal squamous-cell carcinoma patients, and only one study explored the efficacy and adverse event of RT/CRT and ICIs combination for esophageal adenocarcinoma patients. Due to these differing primary efficacy variables, we were unable to prevente the performance/efficacy based upon ESCC and EAC status. An AE subgroup analysis was performed for ESCC, which found that the incidence of grade 3-5 adverse reactions was 54.6% (95% CI: 42.0%-67.1%, I2: 0%) for ESCC. The difference between ESCC and EAC in the efficacy of RT/CRT with ICIs is likely worth further exploration.

In our study, the rate of grade 3-5 adverse reactions and pneumonitis in patients receiving CRT/RT and PD-1 inhibitors was 46.3% (95% CI: 32.0%-60.9%, I^2^: 71%) and 0.8% (95% CI: 0.1%-1.5%, I^2^: 0%). Drugs against PD-L1 were used in two studies and PD-1 in four studies. However, the limited number of studies prevented the further exploration of the differences between PD-1 and PD-L1. A previous study stated that the toxicity profiles of PD-1 and PD-L1 inhibitors in NSCLC patients are similar (54). Gu et al. found that pneumonitis was more frequent when using PD-1 inhibitors for lung cancer, but hepatitis, rash and lipase elevation were more frequent in PD-L1 inhibitors (55). At present, there is no head-to-head study to compare the difference in AEs between PD-1 and PD-L1 inhibitors combined with RT. Although PD-1 inhibitors have been associated with a significantly higher incidence of high-grade immune-related pneumonitis (55–58). The potential mechanism involved in the higher incidence of pneumonitis may be the blockage of PD-1-PD-L2 and induced by PD-1 inhibitors. This blockage assists in the release of cytokines and proliferation of self-reactive T cells, leading to the enhancement of the antitumor effect and AEs (59). Li et al. (58) thought that RT rather than ICIs might be the leading reason for the similar incidence of PD-1 and PD-L1 inhibitors when combined with RT. Thus, the selection of candidate ICIs is recommended, primarily depending on their efficacy rather than the toxicity.

Few studies have explored the optimal schedule for the administration of RT/CRT with ICIs for EC. In our study, we found the incidence of grade 3-5 adverse reactions was 44.2% (95% CI: 33.3%-55.1%, I^2^: 0%) and grade 3-5 pneumonitis was 1.9% (95% CI: 0.1%-5.9%, I^2^: 0%) for patients receiving concurrent and sequential CRT/RT and ICI treatment. The limited number of studies prevented a subgroup analysis for ICIs when used concurrently with RT/CRT and ICIs with sequential RT/CRT. Sihag et al. (60) investigated the safety and feasibility ICIs treatment prior to CRT (followed by surgery after 6 to 8 weeks and then adjuvant ICI therapy for 6 months). However, their primary end point for major complications was 30-days, therefore further is analysis needed to explore the sequencing of CRT and ICIs for EC. There remains no definite conclusion regarding this factor. However, OS was found to be significantly (*P*=0.01) worse for metastatic cancer (80% were lung cancer and 20% were other cancers) patients receiving stereotactic body radiotherapy (SBRT/SRT) after completing immunotherapy (3.6 months) when compared to patients that either received SRT before or concurrently with immunotherapy ICIs (13.0 months) (61). Price, et al. (62) also suggested that treatment with ICIs prior to RT was associated with greater PFS (compared to RT after ICIs) for metastatic NSCLC. But, Lesueur, et al. suggested that there may not be OS or PFS differences based upon whether RT was administered before or during/after ICIs for metastatic NSCLC (63).

It is interesting that some preclinical studies have suggested that the optimal timing may depend on the type of ICI. Anti-CTLA-4 therapies were found to be most effective when given prior to radiation therapy, whereas the optimal timing of anti-OX40 delivery was one day following RT during the post-radiation window of increased antigen presentation (64). Recently, Anscher et al. (65) assessed whether there was an increased risk of serious AEs associated with RT when given within 90 days prior to an ICI. The study utilized 16,835 patients from 68 prospective trials for ICIs that were submitted in initial or supplemental licensing applications in the US Food and Drug Administration (FDA) databases through December 2019. In this pooled analysis, they found that the administration of an ICI within 90 days following RT did not appear to be associated with an increased risk of serious AEs. The RT ≤ 90 patients had slightly numerically higher rates of fatigue, endocrinopathies, and pneumonitis vs the no-RT group. These differences were due to low-grade (grade 1-2) AEs, as there was no difference in grade 3 to 4 AEs between the RT and no-RT groups. Thus, it does appear to be safe to administer an ICI within 90 days of receiving RT.

Based upon the current evidence, the use of RT/CRT combined with ICIs appears promising. Nevertheless, a consensus has yet to be reached on many other features that could contribute to the optimal combination strategy (segmentation mode, dose of radiotherapy, selection of chemotherapy regimen and applicability of biomarkers, etc.). A number of EC clinical trials are ongoing, which investigate the use of with CRT/RT with ICIs, some of which are summarized in **Table 2**.

**Table 2:** Ongoing trials for esophageal cancer with CRT/RT plus ICIs.

Clinical Trial	Target	Agents	Phase	Treatment Groups	Condition	Primary Endpoints	N
NCT03544736 INEC-study	PD-1	Nivolumab	I/II	Cohort A: Nivolumab + palliative RT (20-50 Gy) → Nivolumab Cohort B: Nivolumab + dCRT (RT + CBDCA + PTX) (50.4 Gy) → Nivolumab Cohort C: Nivolumab + Neoadj CRT (RT + CBDCA + PTX) (41.4 Gy) → Nivolumab	EC/GEJ	Safety	30
NCT03278626	PD-1	Nivolumab	I/II	Nivolumab + dCRT (RT + CBDCA + PTX) (50.4 Gy)	TanyN1-3/T3-4N0M0 ESCC	Safety	10
NCT04210115 KEYNOTE-975	PD-1	Pembrolizumab	III	Pembrolizumab + dCRT (RT + 5FU + LOHP/CDDP) (50 Gy) →Pembrolizumab vs. placebo + dCRT (RT + 5FU + LOHP/CDDP) → placebo	Unresectable EC/GEJ	OS EFS	600
NCT02830594	PD-1	Pembrolizumab	II	Pembrolizumab + palliative RT	EC/GEJ	Biomarker	14
NCT02844075	PD-1	Pembrolizumab	II	Pembrolizumab+ Neoadj CRT (RT + CBDCA+ taxane) (41.4 Gy) → surgery → Pembrolizumab	T1N1-2/T2-34aN0-2M0 ESCC	pCR	18
NCT03064490 PROCEED	PD-1	Pembrolizumab	II	Pembrolizumab+ Neoadj CRT (RT+CBDCA+ PTX)	rE/rGEJ AC	pCR	38
NCT04435197 PALACE-2	PD-1	Pembrolizumab	II	Pembrolizumab+ Neoadj CRT (RT + CBDCA + PTX) (41.4 Gy)	cT2-T4aNanyM0 rESCC	pCR	143
NCT05103501	PD-1	Pembrolizumab	II	CRT → surgery → Pembrolizumab + 5FU + CDDP → Pembrolizumab	Stage II/III ESCC	DFS	53
NCT04005170	PD-1	Toripalimab	II	Toripalimab + dCRT (RT + CDDP + PTX) (50.4 Gy) → Toripalimab	Unresectable ESCC	cCRR	42
NCT04844385	PD-1	Toripalimab	II	Toripalimab + dCRT (RT + nab-PTX + NDP + CBP) (60.0 Gy)	Unresectable T2-4NanyM0 ESCC	2-year PFS	83
NCT04888403	PD-1	Toripalimab	II	Toripalimab + Neoadj CRT (RT + nab-PTX + NDP + CBP) (41.4 Gy)	T1-T2N1-N2/T3-4aN0-2M0 ESCC	pCR	45
NCT04437212	PD-1	Toripalimab	II	Toripalimab + Neoadj CRT (RT + CDDP + PTX) (41.4 Gy) → surgery → Toripalimab	T1-4aN1-2/T3-4aN0M0 ESCC	MPR	20
NCT04644250	PD-1	Toripalimab	II	Toripalimab + Neoadj CRT (RT + CBDCA + L-PTX) (41.4 Gy)	T3-4aN0-2M0 ESCC	pCR	32
NCT04177875	PD-1	Toripalimab	II	Toripalimab + Neoadj CRT (RT + CDDP + DTX/nab-PTX) (40 Gy)	T2-3N0-1M0 EC	MPR ORR	44
NCT04821765	PD-1	Tislelizumab	II	Tislelizumab + dCRT (RT + CDDP + nab-PTX) (50-60 Gy) → Tislelizumab	ESCC	LCR	35
NCT03957590	PD-1	Tislelizumab	III	Tislelizumab + dCRT (RT + CDDP + PTX) (50.4 Gy) vs. Placebo + dCRT	Localized ESCC	PFS	316
NCT04776590	PD-1	Tislelizumab	II	Tislelizumab+ Neoadj CRT (RT + CBDCA + nab-PTX) (41.4 Gy)	rESCC	pCR	30
NCT05189730	PD-1	Tislelizumab	II	Tislelizumab + Neoadj CRT (RT + CBDCA + PTX) (40 Gy)	T2-3N0-1/T1-3N2M0 ESCC	pCR Safety	80
NCT04973306	PD-1	Tislelizumab	II/III	Tislelizumab + Neoadj CRT (RT + CBDCA + PTX) (41.4 Gy) vs. Neoadj CRT	cT1b-3N1/cT3-4aN0M0 ESCC	pCR OS	176
NCT04512417	PD-1	Camrelizumab	II	Camrelizumab + palliative RT vs. Camrelizumab	EC	PFS	63
NCT05183958	PD-1	Camrelizumab	II	Camrelizumab + CT (PTX + CBDCA/5FU + CDDP/CAP) → palliative RT → Camrelizumab vs. Camrelizumab + CT (PTX + CBDCA/5FU + CDDP/CAP) → Camrelizumab	ESCC	PFS	118
NCT04404491	PD-1	Camrelizumab	III	Camrelizumab + dCRT (RT + LOHP + CAP) (50-50.4 Gy) vs. Placebo + dCRT	Stage II-IVA ESCC	Safety PFS	240
NCT04426955	PD-1	Camrelizumab	III	Camrelizumab + dCRT (RT + CDDP + PTX) vs. Placebo + dCRT	Localized ESCC	PFS	396
NCT05176002	PD-1	Camrelizumab	I/II	Camrelizumab + Neoadj RT	cT1b-2N+/cT3-4aNanyM0 ESCC	MPR Safety	26
NCT04286958	PD-1	Camrelizumab	II	CRT → Camrelizumab	T1bN+/T2-4N0-2M0 ESCC	PFS	40
NCT04741490	PD-1	Camrelizumab	NA	Surgery → Camrelizumab + RT (45-55 Gy)	T1-4AN0/T1-4AN +M0 ESCC	1-year DFS	20
NCT03940001	PD-1	Sintilimab	I	Sintilimab +Neoadj CRT (RT+CBDCA+PTX) (41.4 Gy)	TanyN+/T3-4NanyM0 ESCC	Safety pCR MPR	20
NCT04212598	PD-1	Sintilimab	II	dCRT/RT → Sintilimab	Stage II/III EC	2-year DFS	40
NCT04514835	PD-1	Sintilimab	II	dCRT (RT + CAP + CDDP) (50-50.4 Gy) → Sintilimab	T1bN+/T2-T4aN0-2M0 ESCC	PFS	44
NCT04602013 IMCORT	PD-1	Sintilimab	II	Sintilimab + dCRT (RT + CDDP + nab-PTX) (60-66 Gy)	Stage II-Iva ESCC	PFS	53
NCT03490292	PD-L1	Avelumab	I/II	Avelumab +Neoadj CRT (RT + CBDCA + PTX)	II/III Stage EC/GEJ	Safety pCR	24
NCT03777813 ARION	PD-L1	Durvalumab	II	Durvalumab + dCRT (RT + 5FU+ LOHP) (50 Gy) → Durvalumab vs. dCRT	Unresectable EC	PFS	120
NCT04054518 DESC	PD-L1	Durvalumab	II	CRT →Durvalumab	EC/GEJ	PFS	22
NCT04851132	PD-L1	Durvalumab	NA	Durvalumab + RT (50.4 Gy)	cT2-4aNanyM0 ESCC	PFS	33
NCT04550260 KUNLUN	PD-L1	Durvalumab	III	Durvalumab + dCRT (RT + 5FU/CAP + CDDP) (50-64 Gy) vs. Placebo + dCRT (RT + 5FU + LOHP) (50-64 Gy)	Stage II-IVA ESCC	PFS	600
NCT02735239 Radio	PD-L1	Durvalumab	I/II	Cohort D: Durvalumab → Durvalumab + Neoadj CRT (RT + CBDCA + PTX) → surgery	Localized EC/GEJ	Safety	75
NCT04568200	PD-L1	Durvalumab	II	Durvalumab + dCRT (RT + CBDCA + PTX) (41.4 Gy) vs. Placebo + dCRT (RT + CBDCA + PTX) (41.4 Gy)	T3/T4bNanyM0 ESCC	pCR	60
NCT02520453	PD-L1	Durvalumab	II	Neoadj CRT → surgery → Durvalumab vs. Neoadj CRT → surgery → Placebo	T3-4N0/T1-4N1-3M0 ESCC	DFS	86
NCT03437200 CRUCIAL	PD-1 & CTLA-4	Nivolumab & Ipilimumab	II	Nivolumab + dCRT (RT + 5FU + LOHP) (50 Gy) → Nivolumab vs. Nivolumab + Ipilimumab+ dCRT → Nivolumab +Ipilimumab	Early stage or locally advanced unresectable EC	1-year PFS	130
NCT03604991	PD-1 & CTLA-4	Nivolumab & Ipilimumab	II/III	Step1: Neoadj CRT (RT + CBDCA + PTX) vs. Neoadj CRT + Nivolumab Step2: adjuvant treatment: Nivolumab vs Nivolumab + Ipilimumab	T1N1-3/T2-3N0-2M0 rE/rGEJ AC	pCR DFS	278
NCT03044613	PD-1 & LAG-3	Nivolumab & Relatlimab	I	Cohort A: Nivolumab → Nivolumab + CRT (RT + CBDCA + PTX) Cohort B: Nivolumab + Relatlimab → Nivolumab + Relatlimab + CRT	II/III Stage EC/GEJ	Safety	32
NCT02962063	PD-L1 & CTLA-4	Durvalumab & Tremelimumab	I/II	Durvalumab + Tremelimumab → Neoadj CRT (RT + CBDCA + PTX) → surgery	TanyN+/T3-4NanyM0 E/GEJ AC	Safety pCR	78
NCT03377400	PD-L1 & CTLA-4	Durvalumab & Tremelimumab	II	Durvalumab/Tremelimumab + dCRT (RT + CDDP + 5FU) → Durvalumab/Tremelimumab	T2-3N0/T1-3N1-3M0 ESCC	PFS	40

N, Number of patients; CRT, Chemoradiotherapy; RT, Radiotherapy; ICIs, Immune checkpoint inhibitors; dCRT, Definitive chemoradiotherapy; CBDCA, Carboplatin; PTX, Paclitaxel; Neoadj, Neoadjuvant; EC, Esophageal cancer; GEJ, Gastroesophageal junction cancer; ESCC, Esophageal squamous cell carcinoma; 5FU, 5-Fluorouracil plus cisplatin; CDDP, Cisplatin; LOHP, Oxaliplatin; OS, Overall survival; EFS, Event-free Survival; pCR, Complete pathologic response rate; rE, Resectable esophageal cancer; AC, Adenocarcinoma; rGEJ, Resectable gastroesophageal junction cancer; rESCC, Resectable esophageal squamous cell carcinoma; DFS, Disease free survival; cCRR, Clinical complete response rate; nab-PTX, Paclitaxel-albumin; NDP, Nedaplatin; CBP, Capecitabine; PFS, Progression free survival; MPR, Major pathological response rate; L-PTX, Paclitaxel liposome; DTX, Docetaxel; ORR, Objective response rate; LCR, Locoregional control rate.

Different radiotherapy dosages and segmentation modes have been reported to have different effects upon the tumor associated immune system. Many pre-clinical studies have suggested that 8-10 Gy in a single fraction appears to generate are more effective anti-tumor when response compared to 2.0 Gy in a single fraction (66–68). A pooled analysis of the PEMBRO-RT and MDACC trials by Welsh et al. found that pembrolizumab when combined with ablative RT (24Gy/3 fractions and 50Gy/4 fractions) had significantly (*P=*<0.05) better ORRs (48% and 54%, respectively) when compared to non-ablative RT (18% ORR with 45Gy/15 fractions) and pembrolizumab alone (20%). It is possible that the higher response rate to ablative RT (compared to non-ablative RT) was due to detrimental effects of non-ablative RT on absolute lymphocyte counts (69).

Other studies have explored the use of 0.5 to 2.0 Gy (with 1 or a few fractions) low-dose radiation therapy (LDRT) to enhance the abscopal response of distant tumors, and to increase the immunogenicity of “cold tumors” (70, 71).The use of hypo-fractionated radiation therapy (HFRT) in combination with ICIs has also been investigated for the induction of antitumor T cell responses. Bilateral mouse tumor models and patients with stage IV NSCLC have demonstrated that a better systemic antitumor response is possible using a triple treatment consisting of LDRT, HFRT and ICIs. Of the nine patients (with metastatic NSCLC) treated with this triple therapy, PR was achieved for three patients and stable disease (SD) for two patients (72). It has been established that it is not feasible to irradiate large segments with HFRT to treat EC. Therefore, LDRT has been proposed for the treatment of advanced EC (metastatic foci), which may warrant further study.

Further consideration should be given to selection of elective nodal irradiation (ENI) because lymph nodes can be a repository for lymphocyte clones against specific antigens, which might affect the curative effects of immunotherapy. It is thought that EC, lung cancer and other thoracic tumor might be more susceptible to radiotherapy due to its effects on circulating lymphocytes that receive different radiation doses as they pass through large blood vessels, the heart and pulmonary circulation. In a preclinical model, Marciscano, et al. showed that stereotactic radiation therapy (SRT) with ENI restrained the adaptive immune response (compared to SRT alone). This effect was associated with the modulation of the chemoattractant and chemokine signature, which led to the reduction of tumor-specific effector T-cell intra-tumoral infiltration and an unfavorable balance between effector T cells and regulatory T cells. Furthermore, ENI was shown to attenuate the combined efficacy of RT and anti-CTLA-4 therapy (73). Another study suggested that tumor draining lymph nodes were enriched with PD-1^+^ T cells, which was associated with prognosis in melanoma following the selective targeting of PD-L1 *via* the induction of effective anti-tumor T-cell responses (74). Therefore, the benefit of omitting ENI could be tested when combining RT/CRT with ICIs for localized/locally advanced disease. However, there is a risk that the omission of ENI could be deleterious for patients with micro-metastases.

There are still some difficulties associated with the identification of patients who would derive the most survival benefit from combination therapy or patients who are more likely to suffer from adverse reactions. PD-L1 expression is a potential biomarker for checkpoint inhibitors in clinical practice. In esophageal cancers, PD-L1 expression and its use for the prediction of immunotherapy efficacy is still controversial. In the KEYNOTE-180 (52) and KEYNOTE-181 (53) trails, patients with CPS PD-L1≥10 seemed to be associated with a slight tumor response improvement when compared to PD-L1 negative patients. Whereas, CheckMate-032 found PD-L1 expression did not correlate with tumor response (75). This indicates that PD-L1 expression is not a perfect marker for immunotherapy outcome.

Current candidate biomarkers also include (76) (1): Cell surface markers: FAS ligands and tumor antigen-specific T cells. (2) Tumor infiltrating lymphocytes (TIL): CD8^+^ TIL, PD-1^+^ CD8^+^ T cells, PD-L1^high^ regulatory T cells (Tregs). (3) Immune related gene expression profiling (GEP): including genes involved with active gamma-interferon (γ-IFN) signaling, T cell cytolytic activity, antigen presentation, chemokine production and adaptive resistance. (4) Tumor mutational burden (TMB). (5) Liquid biopsies for circulating biomarkers: circulating tumor DNA (ctDNA), peripheral blood cells and lymphocyte ratios, such as white blood cells, neutrophil cells, NK cells, monocytes, platelet, lactate dehydrogenase (LDH), neutrophil to lymphocyte ratio (NLR), platelet-to-lymphocyte ratio (PLR) and lymphocyte to monocyte ratio (LMR). (6) Imaging biomarkers: standardized uptake value (SUV), metabolic tumor volume (MTV), and tumor lesion glycolysis (TLG) based upon [18F]- fluoro-2-deoxy-d-glucose (18F-FDG) positron emission tomography (PET). At present, only PD-L1 expression levels on tumor cells have been widely used as a standard predictor to drive anti-PD-1/PD-L1 treatment in the clinic, while multiple other markers detected by genomic, transcriptomic, proteomic and metabolomic analyses are still being investigated and validated.

The combination of RT/CRT and ICIs at any stage of EC (i.e., from early stage to both oligo- and poly-metastatic EC) is offering new hope for the treatment of patients with EC. However, given the dual effect of RT/CRT upon the host immune system the RT/CRT schedule must be optimized whenever a synergistic effect of the combination of RT/CRT and ICIs is expected. To reach this objective, several traditional dogmas about RT might need to be explored in this new therapeutic era, regarding dose, fractionation, target volumes, dose to organs at risk and dose delivery techniques. For EC, how can the timing of RT/CRT and ICIs be used and which chemotherapy regimen may be more effective? Is it better to combine with a PD-1 inhibitor or PD-L1 inhibitor? In the case of sufficient chemotherapy and immunotherapy, can a better therapeutic gain ratio be achieved *via* the reduction of radiation field or radiation dose? Can abscopal response for distant EC be induced by external irradiation of other metastases when combined with ICIs? Additionally, biomarkers to identify the most sensitive population are still being investigated and validated. Thus, both translational and clinical studies are necessary to better understand the mechanisms underlying the immune effects of RT and to provide a strong rationale for this combination.

Our review has some limitations. Firstly, because of the paucity of available data in this field, the number of included studies in our analysis was low. Secondly, most of the included studies in the meta-analysis were single arm clinical trials, we could not compare the advantages and disadvantages of CRT/RT + ICIs and CRT/RT based on a balanced baseline. Thirdly, certain results may contain a high amount of statistical heterogeneity, so subgroup analyze were conducted to examine sources of study heterogeneity. Finally, due to the available data in this field, we could not explore more details regarding the efficacy and safety of ICIs plus RT/CRT, such as the influence of different radiotherapy dose and fractionation, irradiated target volumes, chemotherapy regimen, *etc.* on the relationship of ICIs plus RT/CRT for EC patients.

## 5 Conclusion

In conclusion, this is the first systematic review and meta-analysis to explore the clinical efficacy and safety of immune checkpoint inhibitors when used in combination with radiotherapy/chemoradiotherapy for esophageal cancer patients. Based upon the study contained herein, this mode of therapy appears to be both safe and feasible. However, randomized studies with larger groups of patients need to performed to confirm these results.

## Data availability statement

The raw data supporting the conclusions of this article will be made available by the authors, without undue reservation.

## Author contributions

YZ conceptualized the study. JW, RD, TN, QZ and YL collected the data. JW, RD, TN and FT analyzed the data. JW and YZ wrote the manuscript. All authors contributed to the article and approved the submitted version.

## Funding

This work was supported by the: (1) Science and Technology Fund Project of Guizhou Health Commission (grant no. gzwjkj2020-1-032); (2) Guizhou Province high-level Innovative Talents (grant no. GZSYQCC [2016] 003); (3) LIAN YUN GANG SHI HUI LAN PUBLIC FOUNDATION (HL-HS2020-33); (4) Clinical special of Science and Technology Department of Guizhou Province (grant no. Qiankehechengguo-LC [2021] 015); (5) Health Commission Science and Technology Foundation of Guizhou Province [gzwkj 294 2022-028].

## Acknowledgments

This is a short text to acknowledge the contributions of specific colleagues, institutions, or agencies that aided the efforts of the authors.

## Conflict of interest

The authors declare that the research was conducted in the absence of any commercial or financial relationships that could be construed as a potential conflict of interest.

## Publisher’s note

All claims expressed in this article are solely those of the authors and do not necessarily represent those of their affiliated organizations, or those of the publisher, the editors and the reviewers. Any product that may be evaluated in this article, or claim that may be made by its manufacturer, is not guaranteed or endorsed by the publisher.
