# Supplementary figures and images for: Efficacy and safety of radiotherapy/chemoradiotherapy combined with immune checkpoint inhibitors for locally advanced stages of esophageal cancer: A systematic review and meta-analysis
Source: Front Oncol. 2022 Aug 3;12:887525. doi: 10.3389/fonc.2022.887525 (PMC9381695; doi:10.3389/fonc.2022.887525)

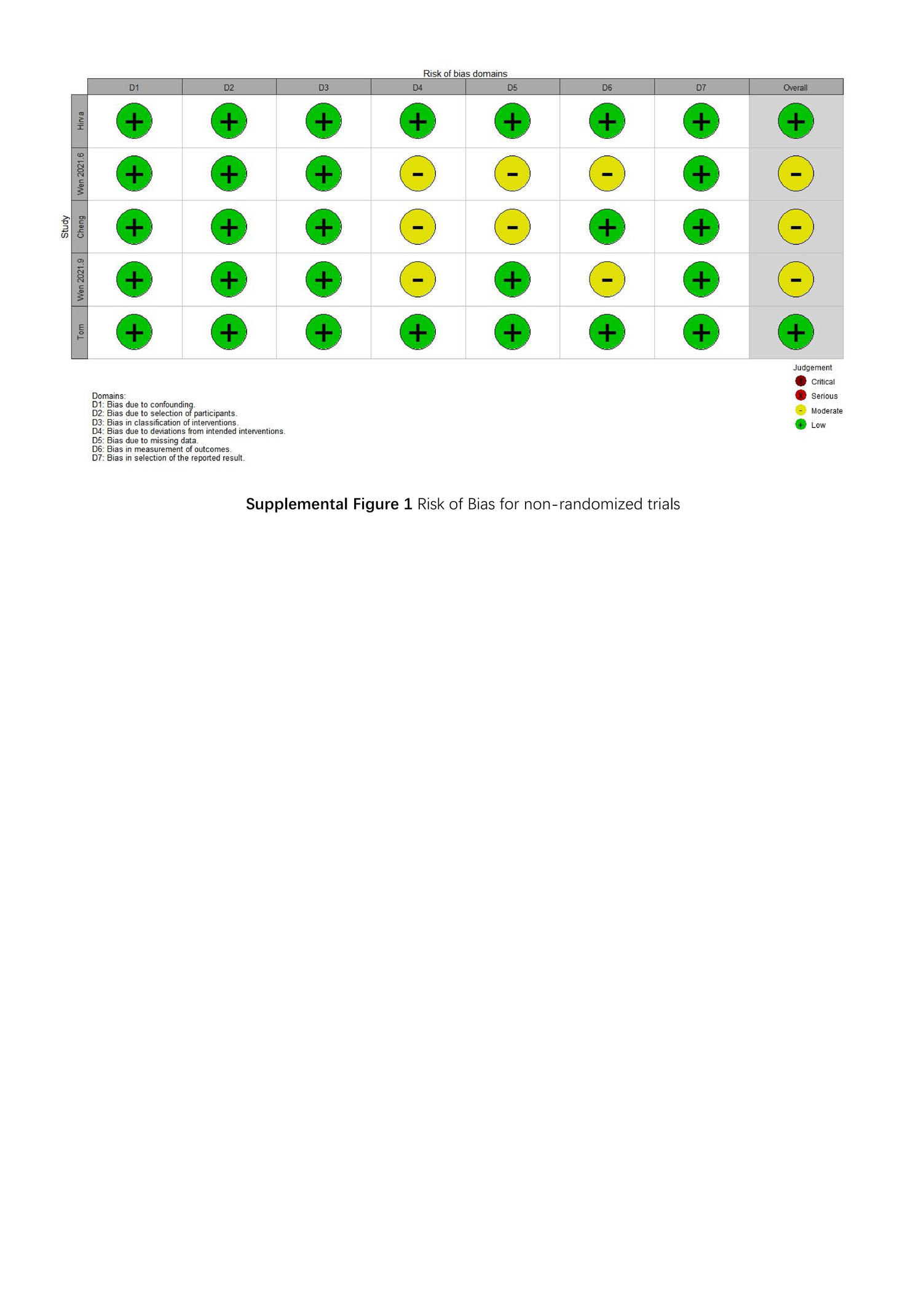

Supplement: Supplementary file 1 [file Image_1.jpeg]

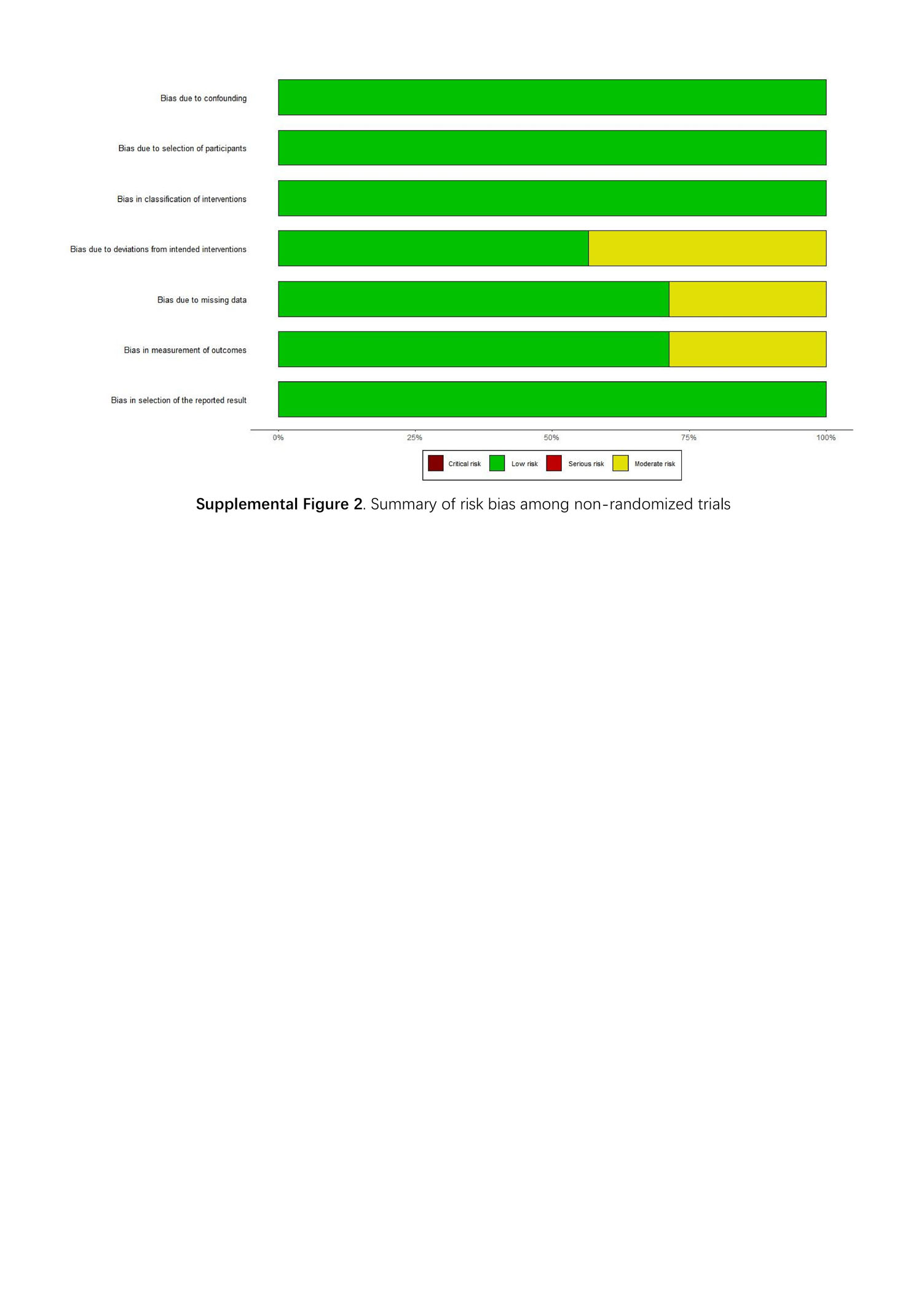

Supplement: Supplementary file 2 [file Image_2.jpeg]

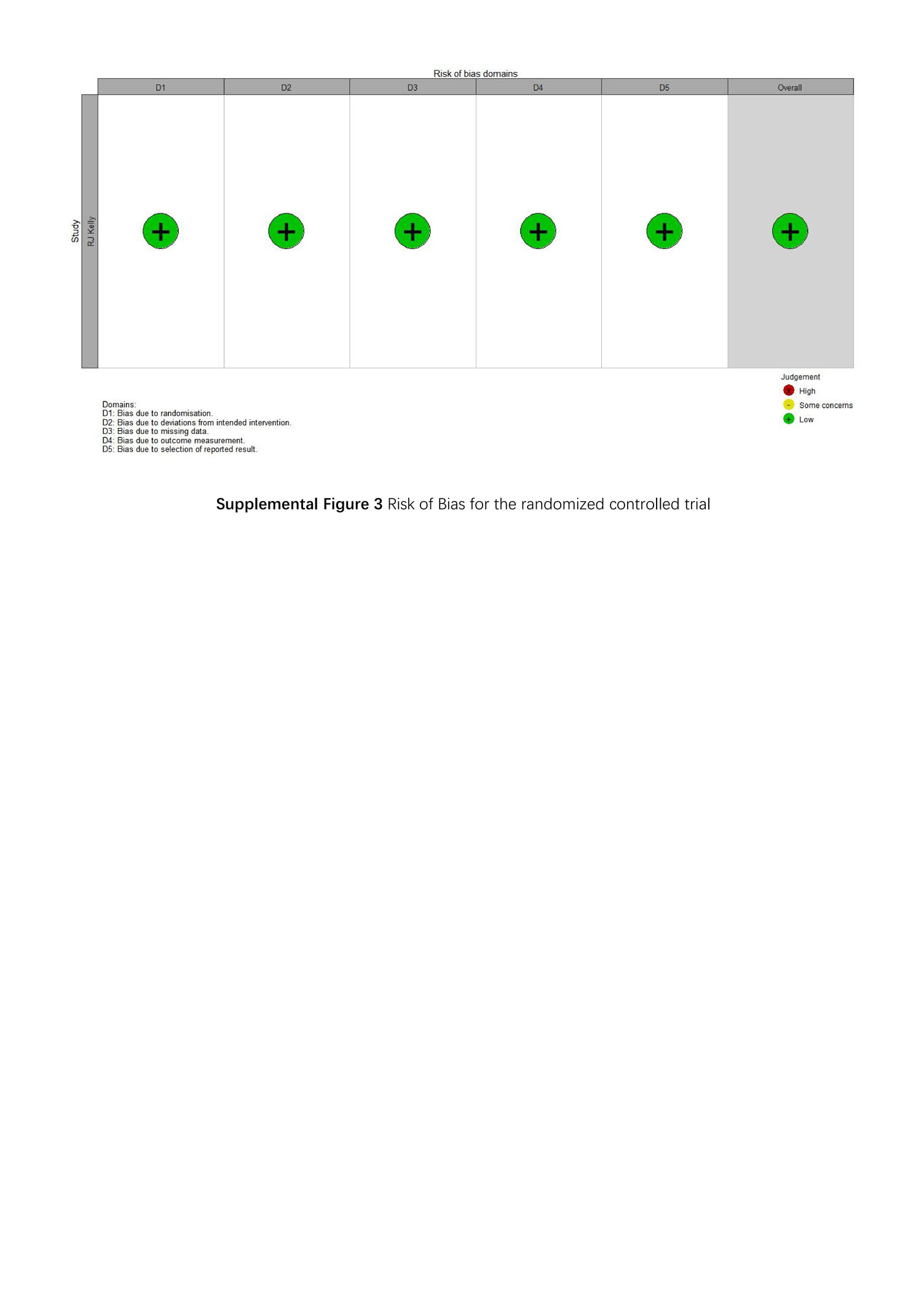

Supplement: Supplementary file 3 [file Image_3.jpeg]

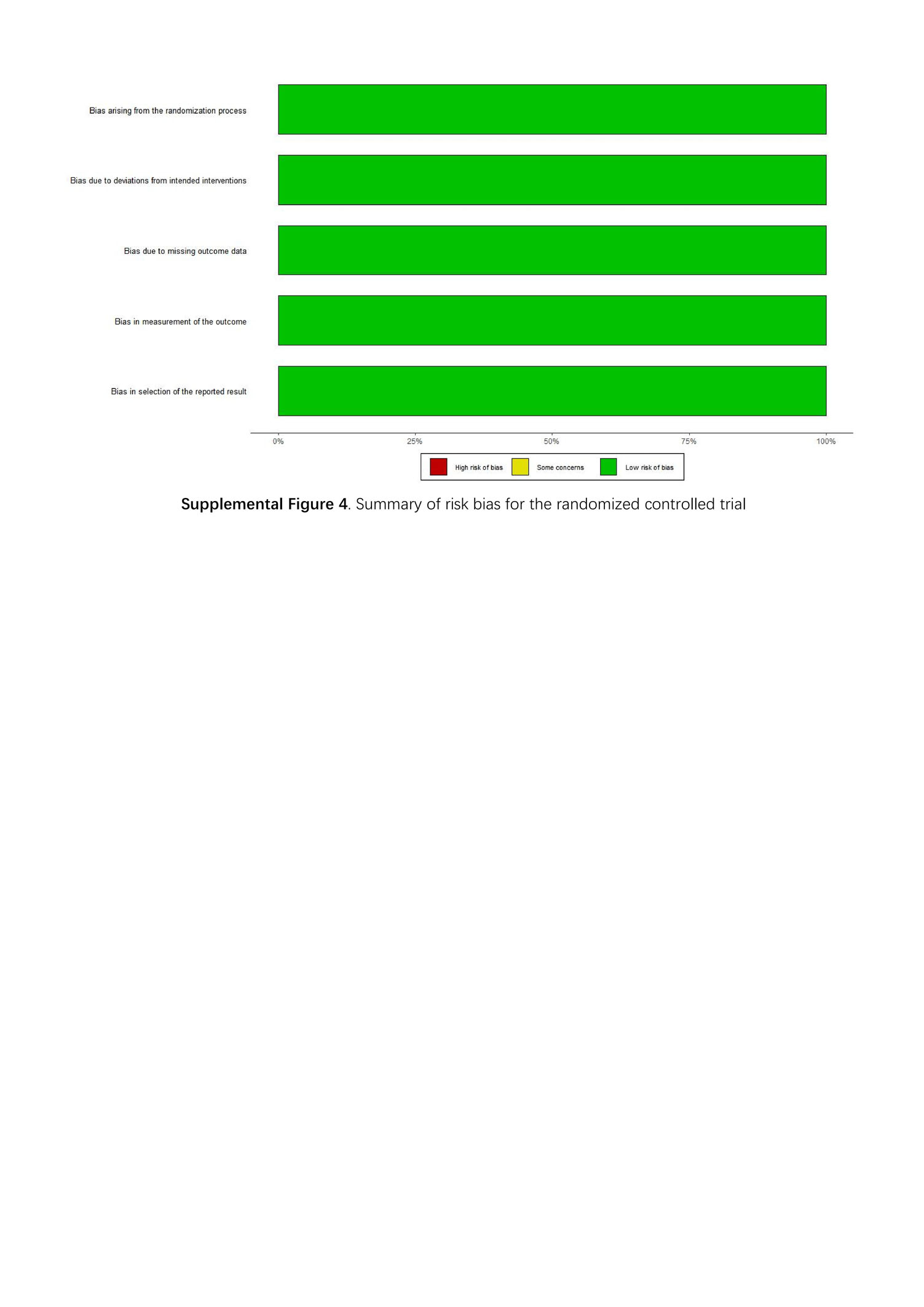

Supplement: Supplementary file 4 [file Image_4.jpeg]

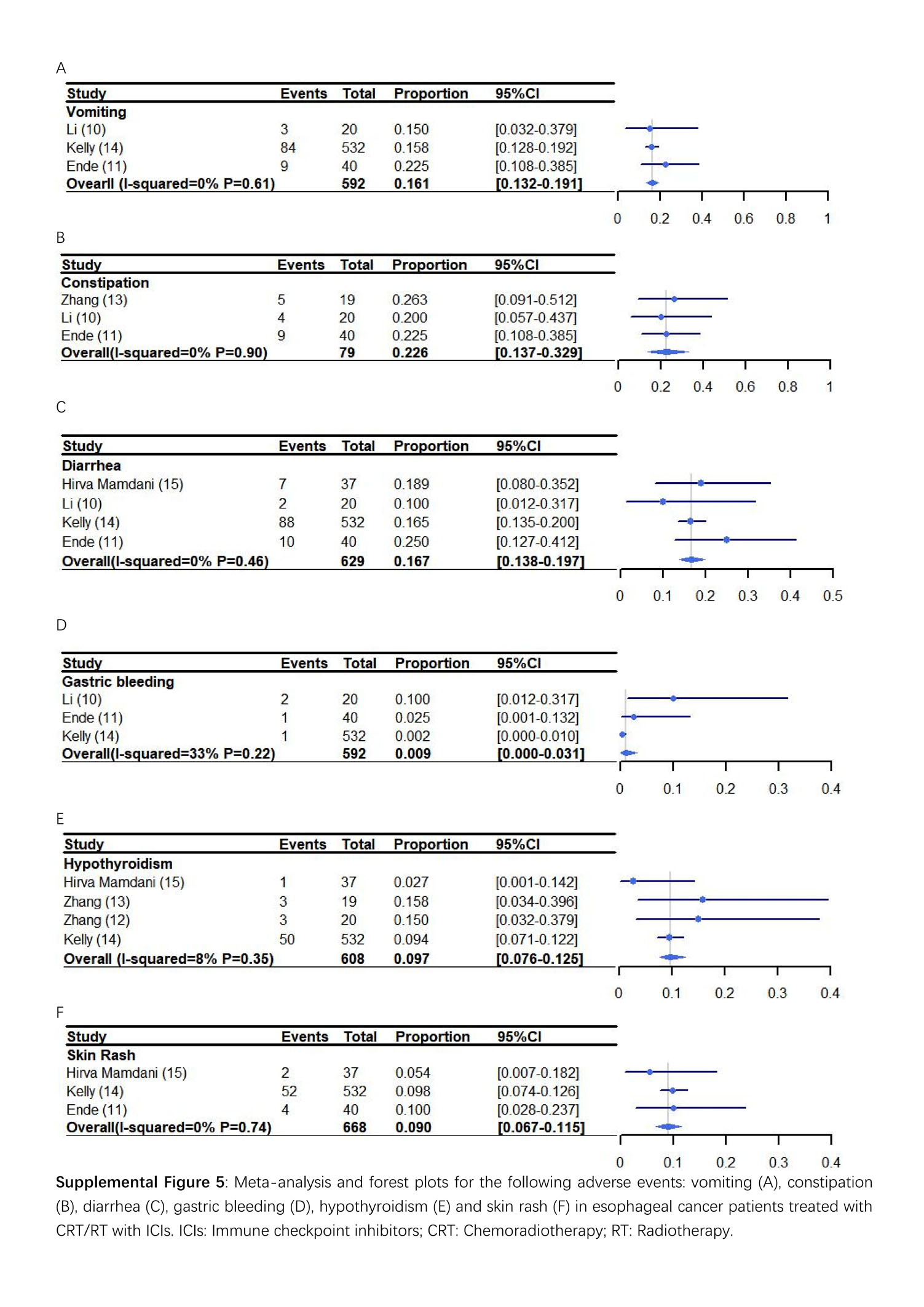

Supplement: Supplementary file 5 [file Image_5.jpeg]
