# Supplementary material for: Efficacy and safety of radiotherapy/chemoradiotherapy combined with immune checkpoint inhibitors for locally advanced stages of esophageal cancer: A systematic review and meta-analysis
Source: Front Oncol. 2022 Aug 3;12:887525. doi: 10.3389/fonc.2022.887525 (PMC9381695; doi:10.3389/fonc.2022.887525)
Supplement: Supplementary file 6 [file Table_1.docx]

**Supplemental Table 1** Publication bias of the meta-analysis

| Subgroup | Begg’s test | Egger’s test |
| --- | --- | --- |
| 1-year OS rate | 0.1172 | 0.1231 |
| 2-Year OS rate | 0.1742 | 0.0510 |
| 2-Year PFS rate | 0.6015 | 0.4592 |
| Grade 3-5 Adverse Events | 0.0558 | 0.1539 |
| Grade 3-5 Adverse Events  (CRT/RT concurrent and sequential ICIs treatment) | 0.1172 | 0.3077 |
| Grade 3-5 Adverse Events (ESCC) | 0.6015 | 0.5038 |
| Grade 3-5 Adverse Events (PD-1) | 0.2786 | 0.0803 |
| Grade 3-5 Adverse Events  (ICIs lasted for more than six months) | 0.1742 | 0.4984 |
| Grade 3-5 Adverse Events  (ICIs combined with CRT) | 0.0770 | 0.3267 |
| Grade 3-5 Pneumonitis | 0.2429 | 0.2375 |
| Grade 3-5 Pneumonitis  (CRT/RT concurrent and sequential ICIs treatment) | 0.6015 | 0.5995 |
| Grade 3-5 Pneumonitis (PD-1) | 0.7021 | 0.7509 |
| Grade 3-5 Pneumonitis  (IT lasted for more than six months) | 0.1742 | 0.3304 |
| Grade 3-5 Pneumonitis  (ICIs combined with CRT) | 0.7947 | 0.5075 |
| Cough | 0.4969 | 0.8112 |
| Pneumonitis | 0.8510 | 0.6902 |
| Esophagitis | 0.6015 | 0.1656 |
| Vomiting | 0.6015 | 0.5969 |
| Constipation | 0.6015 | 0.7846 |
| Diarrhea | 0.4969 | 0.7893 |
| gastric bleeding | 0.1172 | 0.1215 |
| Hypothyroidism | 0.4969 | 0.9280 |
| Skin Rash | 0.1172 | 0.7002 |
